# Changes in respiratory mechanics of artificial pneumothorax two-lung ventilation in video-assisted thoracoscopic esophagectomy in prone position

**DOI:** 10.1038/s41598-021-86554-y

**Published:** 2021-03-26

**Authors:** Yoshinori Tanigawa, Kimihide Nakamura, Tomoko Yamashita, Akira Nakagawachi, Yoshiro Sakaguchi

**Affiliations:** 1 Surgical Center, Saga Medical School Hospital, Faculty of Medicine, Saga University, 5-1-1 Nabeshima, Saga city, Saga, 849-8501 Japan; 2 Intensive Care Unit, Faculty of Medicine, Saga Medical School Hospital, Saga University, Saga, Japan; 3 Department of Anesthesiology and Critical Care Medicine, Faculty of Medicine, Saga Medical School Hospital, Saga University, Saga, Japan

**Keywords:** Medical research, Oncology

## Abstract

We aimed to clarify the changes in respiratory mechanics and factors associated with them in artificial pneumothorax two-lung ventilation in video-assisted thoracoscopic esophagectomy in the prone position (PP-VATS-E) for esophageal cancer. Data of patients with esophageal cancer, who underwent PP-VATs-E were retrospectively analyzed. Our primary outcome was the change in the respiratory mechanics after intubation (T1), in the prone position (T2), after initiation of the artificial pneumothorax two-lung ventilation (T3), at 1 and 2 h (T4 and T5), in the supine position (T6), and after laparoscopy (T7). The secondary outcome was identifying factors affecting the change in dynamic lung compliance (Cdyn). Sixty-seven patients were included. Cdyn values were significantly lower at T3, T4, and T5 than at T1 (p < 0.001). End-expiratory flow was significantly higher at T4 and T5 than at T1 (p < 0.05). Body mass index and preoperative FEV_1.0%_ were found to significantly influence Cdyn reduction during artificial pneumothorax and two-lung ventilation (OR [95% CI]: 1.29 [1.03–2.24] and 0.20 (0.05–0.44); p = 0.010 and p = 0.034, respectively]. Changes in driving pressure were nonsignificant, and hypoxemia requiring treatment was not noted. This study suggests that in PP-VATs-E, artificial pneumothorax two-lung ventilation is safer for the management of anesthesia than conventional one-lung ventilation (UMIN Registry: 000042174).

## Introduction

Esophageal cancer is the eighth most common cancer and sixth most common cause of cancer-related mortality. Surgery for esophageal cancer is a highly invasive procedure; postoperative respiratory complications, which is the most common cause of mortality, occur in 20–30% of the cases, and the perioperative mortality rate is 15% higher than that of other cancers^1–4^.

In recent years, video-assisted thoracoscopic esophagectomy (VATS-E) has been introduced as an alternative to right thoracotomy to reduce invasion and provide visual magnification under the microscope^5,6^.

VATS-E in the left decubitus position (LP-VATS-E) was widely performed until recently; however, despite this position, a skillful technique to secure the field of view in the mediastinal position was required. The prone position video-assisted thoracoscopic esophagectomy (PP-VATS-E), which enables easier deployment of the operative field around the longitudinal cage than LP-VATS-E, was first reported by Cushieri in 1994^7^ and has become well known since Palanivelu's report of 130 cases in 2006^8^. This method facilitates vascular and nerve preservation and mediastinal lymph node dissection by shifting the lungs and the leachate ventrally according to gravity without applying excessive pressure and drainage to the diseased lung to maintain a good visual field. Although the operative time between the LP and PP groups was not different as reported by Kuwabara et al.^9^, the PP group had lesser intraoperative blood loss, lower incidence of complications of Clavien–Dindo grade III or higher, and shorter hospital stay. PP-VATS-E was less invasive and an independent risk factor for postoperative pulmonary complications. However, variations in respiratory dynamics during the perioperative period due to body position were not addressed^9^.

Respiratory management of VATS-E has been performed at many centers for one-lung ventilation using a bilumen intubation tube or a bronchial blocker during thoracic operations. However, the risk of edema, injury, and recurrent nerve damage to the glottis, trachea, mainstem bronchus, and peri-pharyngeal tissues is increased when using a bilumen intubation tube, and difficulty in positioning the balloon catheter is experienced when using a bronchial blocker^10^.

At our institution, PP-VATS-E has been performed since 2014 with artificial pneumothorax in the operative right lung in conjunction with two-lung ventilation using a single lumen tube for respiratory management. VATS-E with this ventilation technique has been reported to be superior to one-lung ventilation in terms of reduced postoperative oxygenation and blood loss^11^, shorter operative time^12,13^, easier capture of the operative field around the recurrent nerve and lymph node awakening^13,14^, and improved perioperative outcomes, including postoperative intensive care unit admission and hospital stay^15–17^.

However, in the prone position, the improvement in ventilatory blood flow ratio due to hypoxic pulmonary vasoconstriction during one-lung ventilation may not be as effective as in the left decubitus position, and pneumothorax may reduce compliance in the collapsed lung^18^. There are limited studies investigating the respiratory status of patients undergoing thoracoscopic esophagectomy in the prone position and during artificial pneumothorax. In this study, we hypothesized that changes in respiratory mechanics caused by artificial pneumothorax and two-lung ventilation in PP-VATs-E would have no significant adverse effects on the body and that this would be a safe method of anesthesia management.

## Methods

### Patient selection

This retrospective observational study was performed in the Saga University Hospital, Japan. The study was conducted in accordance with the Declaration of Helsinki and the ethical guidelines for epidemiological research of the Ministry of Education, Culture, Sports, Science and Technology. The protocol was approved by the Ethics Committee of Saga University Hospital and is available on the website of Saga University Clinical Research Center (Saga, Japan, Number 20180104, Date 20180104). By the Ethics Committee of Saga University Hospital, the need for informed consent was waived given the retrospective nature of the study.

A registry of patients who underwent VATS-E performed by one surgeon and the same anesthesia machine (FLOW-i; GETINGE AB, Gothenburg, Sweden) was initiated in November 2015 to September 2018.

The patients’ inclusion criteria were (1) American Society of Anesthesiologists (ASA) grade 1–3, (2) body mass index > 15 and < 30 kg/m^−2^ (3) diagnosis of esophagectomy cancer without remote metastasis lesion such as brain or lung metastases. Patients who had moderate to severe valvular heart disease, left ventricular ejection fraction < 50%, a history of chronic obstructive pulmonary disease, difficulty in intrathoracic manipulation (scoliosis, contracture, and high adhesions) and communication due to dementia or mental illness, had hypopharyngectomy complications, were unable to lie down, underwent open chest surgery, non-radical surgery, and emergency esophagectomy were excluded. Patient and intraoperative data were extracted from the electronic medical records and anesthesia records.

### Anesthetic techniques

In all cases, a central venous catheter was placed through the right subclavian vein a day before the operation. An electrocardiogram, pulse oximeter, and noninvasive blood pressure monitor were instituted after the patient was brought to the operating room. Propofol 1.0 mg kg^−1^, remifentanil 0.3–0.5 μg kg^−1^ min^−1^, and rocuronium 0.6 mg kg^−1^ were used for induction of anesthesia, and a single lumen tube with a 7.0–8.0 mm inner diameter spiral (Parker Endotracheal tube; Next Japan Medicalnext Corporation, Tokyo, Japan) was used to perform tracheal intubation. After induction, an open arterial pressure line was secured in the right radial artery and connected to a FloTrac/Vigileo (Edwards Lifesciences Corporation, Irvine, USA) for measurement. Intraoperatively, anesthesia was maintained with sevoflurane 1.0–1.5%, remifentanil 0.2–0.5 μg kg^−1^ min^−1^, with the addition of fentanyl and rocuronium as appropriate. With respect to the ventilation conditions, pressure control ventilation was used. Ventilation settings were TV < 8 mL/kg^−1^, PEEP 5 cm H2O, and respiratory rate 10–12 breaths/min^−1^. Ventilation, including inspiratory oxygenation, was adjusted so that airway pressure was less than 30 cm H_2_O and SpO_2_ was greater than 90%. After induction of anesthesia and measurement of the assessed variables, the patient was repositioned to the supine position, the trunk was immobilized, and the assessed variables were measured again with the same ventilator settings as in the supine position. After the start of the operation, the four-port supply mirror technique used in artificial pneumothorax surgery was used. The observation port was placed in the posterior axilla of the fifth intercostal space with a 10 mm scope. A CO_2_ pneumothorax was started at 8–10 mmHg using a trocar system to the same site^19,20^, and an artificial pneumothorax was maintained using ENDOPATH XCEL OPTIVIEW (Johnson and Johnson K. K., Tokyo, Japan). For further intrathoracic procedures, ports were placed at the subscapular end of the 9th intercostal space, the posterior axilla of the 7th intercostal space, and the middle axilla of the 3rd intercostal space, respectively. The basic settings for artificial pneumothorax were tidal volume (TV) < 6 mL/kg^−1^, positive end-expiratory pressure (PEEP) 5–7 cm H_2_O, and respiratory rate 15–20 breaths/min^−1^. The thoracotomy was a single operation performed by two surgeons and a scopist. The left mediastinal pleura was preserved as much as possible and there was no physical drainage to the lungs. The artificial pneumothorax was discontinued after completion of the intrathoracic procedure, and the patient was then repositioned to the supine position for the intra-abdominal procedure. Monitoring was performed until 15 min after the start of the laparoscopic laparotomy with pneumoperitoneum. The ventilator settings at the end of the artificial pneumothorax were TV < 8 mL/kg^−1^, PEEP 5 cm H_2_O, and respiratory rate 10–12 breaths/min^−1^. The artificial pneumothorax pressure was 8–10 mmHg.

### Study outcome

The primary endpoint was the change in the respiratory mechanics measured after tracheal intubation (T1), in the prone position (T2), after initiation of artificial pneumothorax two-lung ventilation (T3), after 1 and 2 h (T4 and T5) of the initiation of artificial pneumothorax two-lung ventilation, in the supine position (T6), and after initiation of laparoscopy (T7). The measures of respiratory mechanics included dynamic lung compliance (Cdyn), end-expiratory flow rate (Vee), end-expiratory partial pressure of CO_2_ (EtCO_2_), peak inspiratory pressure (PIP), and driving pressure (ΔP = highest airway internal pressure-PEEP). Measurements at each point were recorded on an electronic anesthesia recording system (Mirrel-CVW 500; FUKUDA DENSI, Tokyo, Japan), with data measured at every second during the 5 min before and after.

Secondary endpoints were pH, PaCO_2_, PaO_2_, P/F ratio, and related factors affecting the change in Cdyn (T1-T4/T1) obtained from blood gas tests at T1, T2, T4, and T6. Data regarding relevant factors, including sex, age, height, weight, preoperative pulmonary function tests (%VC, FEV_1.0%_), operative time, anesthesia time, pneumothorax time, intraoperative fluid, transfusion balance, intraoperative urine volume, intraoperative blood loss, intraoperative blood transfusion, staging, and minimum intraoperative PaO_2_/FiO_2_ ratio (P/F ratio), were extracted.

### Statistical analysis

Considering the variables describing the patients’ characteristics, the median and range are reported for the continuous variables, and absolute and relative frequencies are reported for the categorical variables. For changes in respiratory mechanics, the values at each measurement point were compared using the Dunnett test with the T1 value as a control. In addition, T3, T4, and T5 were compared using the repeated measures ANOVA test and PostHoc test (Turkey HSD test) for time analysis since there was no change in the accepted protocol for time intervention.

The percentage change of Cydn was defined as (T1-T4)/T1 and categorized in two groups (acceptable / unacceptable value groups) using a median cutoff point. We used variable selection to select the factors that predict the change in Cdyn. Cdyn(T1-T4/T1) was used as the target variable, and the unadjusted odds ratio (OR) was estimated regardless of patient characteristics, and the adjusted OR was estimated using a multiple logistic model that included patient characteristics including age, BMI, and FEV_1.0_% as confounders. Next, thoracic surgery time was examined in a stepwise model as a variable that should necessarily be included. When selecting variables in the stepwise method, Akaike's Information Criterion was used as an indicator for model selection.

The ORs and 95% confidence intervals (CIs) were determined to describe associations. All statistical tests were two-sided; a p value < 0.05 was considered statistically significant. The sample size estimate yielded a value of 67 patients. The input would be n = 67, significance level = 0.05, and effect size = 0.4. Using Cohen’s paired-t-test power calculation formula and assuming a two-sided test using the aforementioned parameters, a power of 0.897 would be attained. Statistical analyses were performed using JMP version 13.1.0 (SAS Institute Japan, Tokyo, Japan).

## Results

**Figure 1:**
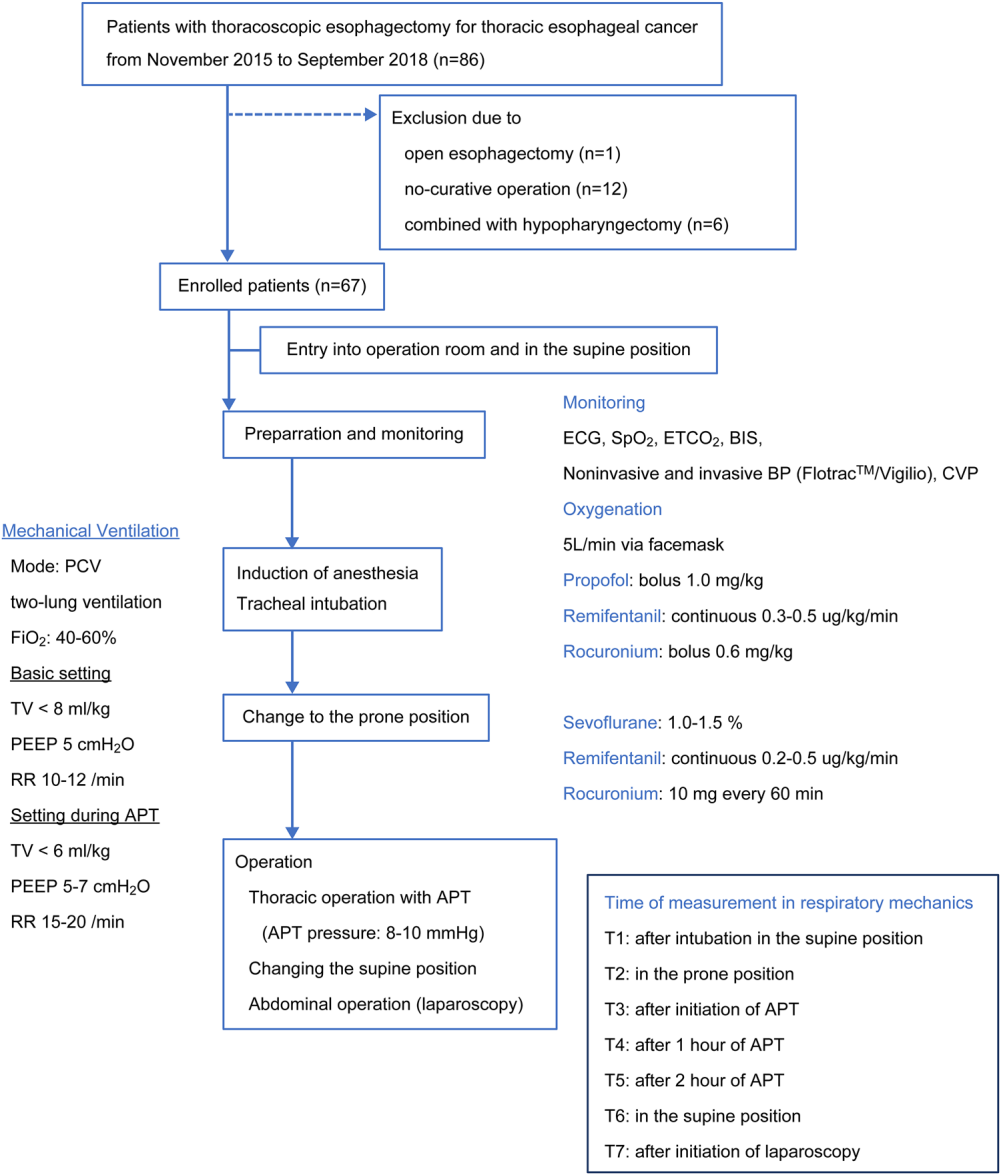
Summary of the longitudinal study design and consort diagram of patients enrolled. BIS: bispectral index, BP: blood pressure, ECG: electrocardiogram, ETCO_2:_ end-tidal CO_2_ monitoring, FiO2: fraction of inspired oxygen, PEEP: positive end-expiratory pressure, PCV: pressure control ventilation, APT: artificial pneumothorax, TV: tidal volume, RR: respiratory rate.

A total of 19,374 patients underwent surgical treatment, for various ailments, in the Saga University Hospital’s operation room during the study period, and among them 86 patients were treated with VATS-E for esophageal cancer. Patients were excluded in case of open esophagectomy (n = 1), no curative operation (n = 12), or total laryngopharyngoesophagectomy (n = 6). Therefore, we retrospectively analyzed data of 67 patients (Fig. 1).

The background of the 67 patients in whom PP-VATS-E could be completed is shown in Table 1. The median operative time, anesthesia time, and pneumothorax time were 539 min, 654 min, and 213 min, respectively.

**Table 1:** Patient characteristics and operative outcomes.

Characteristics	Variables^a^ (n = 67)
Sex	
Male	52 (77.6)
Female	15 (22.3)
Age (years)	70 (65–76)
Height (cm)	162 (157–167)
Weight (kg)	55 (49–61)
BMI	21.3 (18.9–23.8)
ASA score	
I	9 (13.4)
II	45 (67.1)
III	13 (19.4)
Smoking	
Yes/No	46 (68.6)/21 (31.3)
Pulmonary function	
%VC ≦ 80%	3 (4.4)
FEV_1.0%_ ≦ 70%	21 (31.3)
Operation time (min)	539 (461–684)
Anesthesia time (min)	654 (555–780)
Thoracic operation time (min)	213 (187–285)
Intraoperative infusion (ml/kg/h)	5.5 (4.2–6.4)
Intraoperative Urine volume (ml/kg/h)	1.4 (0.9–2.4)
Intraoperative blood loss (ml)	110 (51–285)

ASA, American Society of Anesthesiology; BMI, body mass index; VC, vital capacity; FEV, forced expiratory volume.

^a^Values in parentheses are percentages unless indicated otherwise; continuous values are expressed with median (interquartile range).

**Figure 2:**
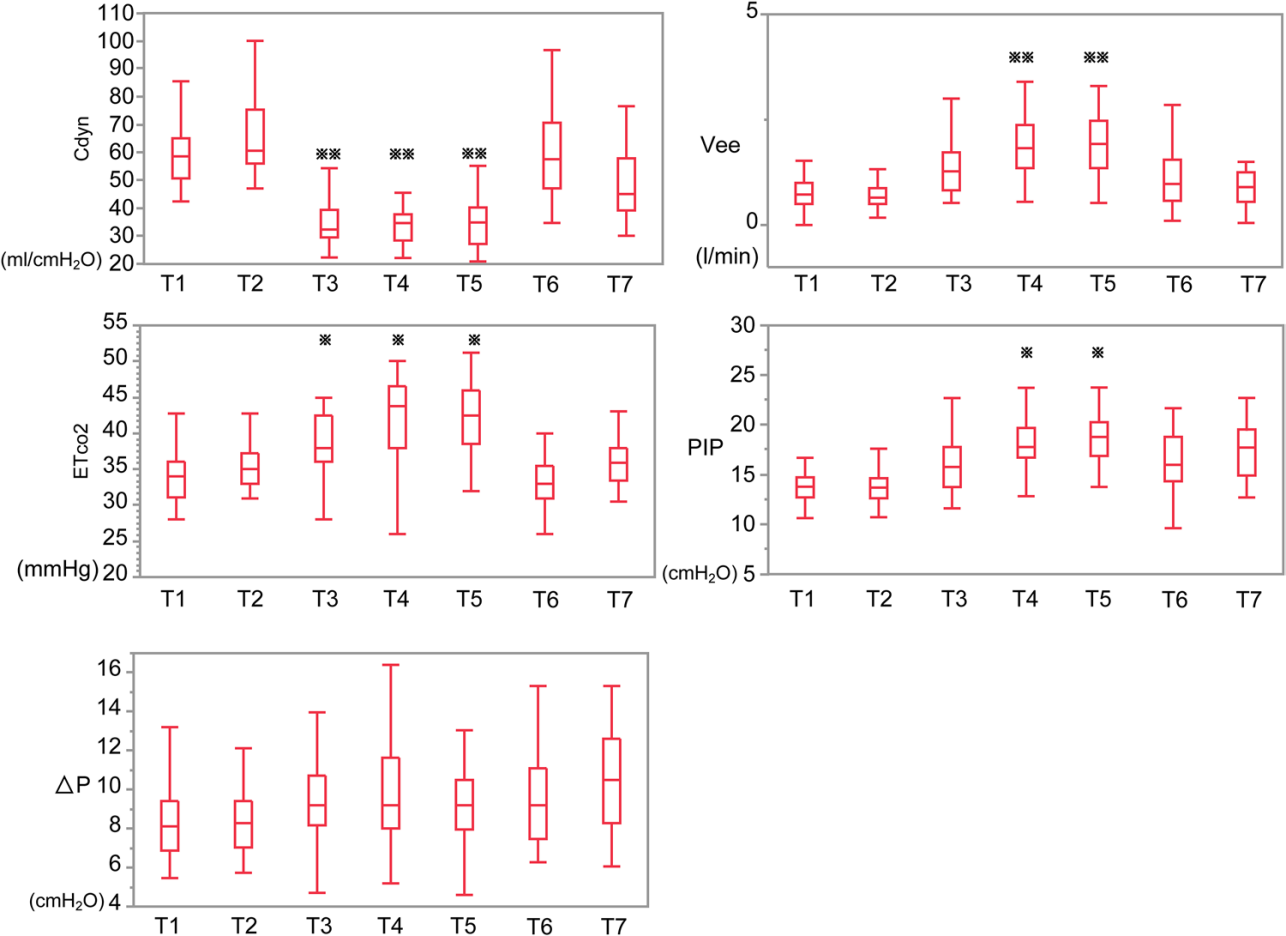
Intraoperative changes in respiratory parameters under two-lung ventilation. The horizontal bars indicate the median values. The vertical bars indicate the range, and the horizontal boundaries of the boxes represent the first and third quartiles. *p < 0.05, **p < 0.01 significantly different from T1. T1: supine position, T2: prone position, T3: 10 min, T4: 1 h, and T5: 2 h after the start of artificial pneumothorax, T6: supine position after the end of artificial pneumothorax, T7: 10 min after the start of laparoscopic surgery. Cdyn: dynamic compliance, Vee: Velocity of end-expiratory flow, ETco2: end-tidal carbon dioxide concentration, ΔP: driving pressure.

The results of the analysis of changes in the primary endpoint of respiratory mechanics are shown in Fig. 2. Cdyn was significantly lower at T3, T4, and T5 than at T1 (p < 0.05). Vee and PIP were significantly higher at T4 and T5 than at T1 (p < 0.05). EtCO_2_ was significantly higher at T3, T4, and T5 than at T1 (p < 0.05). Change in driving pressure when artificial pneumothorax two-lung ventilation was performed was nonsignificant compared to the supine or prone position without artificial pneumothorax. The results of the analysis of changes in the primary endpoint of respiratory mechanics during artificial pneumothorax are shown in Table 2. PIP during artificial pneumothorax showed a significant increase between T3 and T5, and a significant tendency to increase between T3 and T4.

**Table 2:** Change in respiratory parameters during APT (T3, T4, and T5).

	Repeated ANOVA			Post-hoc results analyzed by Turkey HSD test				
	F	DF	p value	Condition A	Condition B	A-B	p value	95% CI
Cdyn	0.50	(3, 138)	0.61	T3	T4	1.36	0.82	−3.96 to 6.67
				T5	T4	2.27	0.58	−3.17 to 7.72
				T5	T3	2.29	0.93	−4.53 to 6.36
Vee	3.31	(3, 138)	0.61	T4	T3	0.55	0.53	−0.65 to 1.74
				T5	T4	0.78	0.29	−0.45 to 2.00
				T5	T3	1.32	0.03	0.09 to 2.54
EtCO2	3.57	(3, 138)	0.03	T4	T3	3.09	0.07	−0.19 to 6.38
				T5	T4	0.26	0.98	−3.13 to 3.66
				T5	T3	3.36	0.05	−0.04 to 6.75
PIP	8.28	(3, 138)	0.00	T4	T3	2.17	0.05	−0.01 to 4.35
				T5	T4	1.62	0.20	−0.62 to 3.85
				T5	T3	3.79	0.00	1.56 to 6.02
ΔP	0.57	(3, 138)	0.57	T4	T3	0.63	0.54	−0.78 to 2.03
				T4	T5	0.27	0.83	−1.09 to 1.80
				T5	T3	0.35	0.89	−1.09 to 1.80

DF, degree of freedom; OR, odds ratio; CI, confidence interval; ETCO_2_, end-tidal CO_2_ monitoring, Cdyn: change in dynamic lung compliance; Vee, Velocity of end-expiratory flow; ETco2, end-tidal carbon dioxide concentration; ΔP, driving pressure; APT, artificial pneumothorax; PIP, peak inspiratory pressure.

The results of the secondary endpoints of arterial blood gas testing are shown in Table 3. P/F ratio was higher at T2 and significantly lower at T4 than at T1. PaCO_2_ was significantly higher at T4 than at T1. The results of the analysis of the factors affecting the changes in Cdyn during supine pneumothorax are shown in Table 4. The multivariate analysis showed that body mass index (BMI) and preoperative FEV_1.0%_ were factors that significantly affected respiratory mechanics (p = 0.01 and 0.03, OR = 1.29 and 0.20, respectively).

**Table 3:** Results of the arterial blood gas analysis of the 67 patients included in the study.

Items	T1	T2	T4	T6
PaO_2_ (mmHg)	248 (6.6, 205–280)	252 (7.5, 213–287)	131** (5.6, 120–142)	202* (6.0, 190–214)
P/F ratio	376 (15, 375–422)	455 (13, 392–520)	211** (9, 193–229)	374 (11, 352–397)
PaCO_2_ (mmHg)	42 (0.6, 39–45)	40 (0.6, 36–42)	56* (1.2, 53–58)	41 (0.6, 40–44)
pH (median)	7.32 (0.006, 7.37–7.41)	7.46 (0.007, 7.43–7.47)	7.32* (0.007, 7.31–7.34)	7.40 (0.005, 7.38–7.41)

The Mann–Whitney U test was used to analyze continuous variables.

Compared with T1, *p < 0.05, **p < 0.01.

*PaO*_*2*_, oxygen in arterial blood; *IQR,* inter quartile range; *PaCO*_*2*_, carbon dioxide in arterial pressure; *P/F ratio,* oxygen in arterial blood/fraction of inspired oxygen.

Continuous values are expressed with median (standard Error, interquartile range).

**Table 4:** Univariable and multivariate logistic regression analysis of the risk factors for Cdyn changes.

Risk Factor	Unadjusted OR	*P* value	Adjusted OR	95% CI	*p* value
Sex (Male)	3.80	0.084	6.223	1.82–10.62	0.007
Age	0.12	0.524	–	–	–
Stage	1.99	0.256	–	–	–
BMI	0.20	0.075	1.29	1.03–2.24	0.010
FEV_1.0%_	0.15	0.082	0.20	0.05–0.44	0.034
P/F ratio	0.20	0.752	–	–	–
Blood transfusion	0.05	0.085	–	–	–
Smoking	0.002	0.780	4.44	0.65–9.53	0.084
Thoracic operation time	6.00	0.218	–	–	–

P/F ratio, oxygen in arterial blood/fraction of inspired oxygen in the preoperative outpatient clinic; BMI, body mass index; FEV, forced expiratory volume; OR, odds ratio; CI, confidence interval; Cdyn: change in dynamic lung compliance.

## Discussion

To the best of our knowledge, this is the first study consisting of 67 patients who underwent PP-VATS-E for esophageal cancer focusing on the changes in respiratory mechanics during artificial pneumothorax two-lung ventilation.

Changes in driving pressure were nonsignificant, although Cdyn values were significantly lower and end-expiratory flow was higher with artificial pneumothorax two-lung ventilation than in the supine position. In line with previous reports, we found hypercarbon dioxemia during thoracic maneuvers, non-severe decrease in transcutaneous O_2_ saturation or P/F ratio^19–22^, and no case that needed conversion to one-lung ventilation^23^.

In the prone position, Pressure Redistribution Urethane Foam (Soft-nurse; ALCARE, Tokyo, Japan) was used to prevent an increase in contact pressure. Nevertheless, fixation of patients was necessary to prevent them from falling off the bed; hence, despite the use of Pressure Redistribution Urethane Foam, there was a risk of diaphragmatic motion restriction, which may have led to reduction in thoracic compliance.. However, in this study, Cdyn and Vee were largely unaffected by the negative effects, and the P/F ratio showed a trend toward improvement. The effect of prone position has been reported to improve prognosis for severe acute respiratory distress syndrome, and its respiratory physiological benefit is the correction of ventilatory and pulmonary blood flow inequalities^24–26^. In the supine position, the dorsal lung is hypoventilated and atelectasis is formed in comparison to the ventral lung, whereas the prone position increases functional residual capacity and homogenizes the size of the ventral and dorsal alveoli due to the effects of gravity and shape matching^27,28^. In addition, pulmonary blood flow is less affected by gravity, and the distribution of pulmonary blood flow is dorsally dominant, which improves the difference in the ratio of ventilated blood flow between the dorsal and ventral sides of the lung. In this study, Cdyn did not decrease and PIP did not increase in the prone position, and the P/F ratio showed a trend of improvement, suggesting that oxygenation may be improved by homogenizing the diastolic pressure on the ventral and dorsal alveoli and improving the ventilatory blood flow ratio.

With regard to the ventilation method, it has been reported that the median P/F ratio was less than 150 in both the left decubitus and prone positions when conventional one-lung ventilation was used during thoracic maneuvers^16,18,29^, and the median P/F ratio was lower in the supine position. In addition, since hypoxic pulmonary vasoconstriction is less likely to occur in the prone position^17^, cases of severe hypoxemia have also been reported^18^. However, in this study, Cdyn during chest maneuvers that underwent artificial pneumothorax two-lung ventilation in the prone position was < 40 ml/cm H_2_O, equivalent to a P/F ratio of 100–170^11^, but the P/F ratio was maintained above 200, and no cases of critical hypoxemia were observed.

Since Cdyn is measured in the presence of airflow, it is more sensitive to airway resistance and respiratory frequency as well as to the elastic properties of the lung than is static lung compliance (Cst), which is measured when the airflow is paused. In this study, the Vee value, which usually approximates to 0 L min^−1^, remained significantly higher during artificial pneumothorax, suggesting an increase in the airway resistance. Considering the fact that many patients had to breathe more than 20 breaths to ensure minute ventilation, the Cdyn measured during artificial pneumothorax was lower than the actual value, which may be more pronounced in case of preoperative obesity or obstructive disorders that may affect the airway system.

Essentially, an accurate measure of the status of functional lung areas involved in ventilation is the compliance of the respiratory system (CRS), which is calculated from the lung compliance and thoracic compliance. The CRS was difficult to measure in this study because the Cdyn measured in this study was lung compliance, and the measurement of thoracic compliance requires the measurement of intrathoracic pressure using a dedicated esophageal balloon catheter. However, driving pressure (ΔP), which is the single-volume divided by CRS (ΔP = tidal volume/CRS), can be clinically measured by the pressure gradient between peak airway pressure and PEEP, which is a direct measure of the stretching pressure (stress) and extension to the functional lung area involved in ventilation (strain). Increased mortality and pulmonary complications have been reported in ARDS cases exceeding 15–20 cm H_2_O of ΔP^30,31^. In this study, a constant pneumothorax pressure was added to the right intrathoracic pressure during thoracic maneuvering, but the increase in ΔP was minimal and no cases exceeding 16 cm H_2_O were observed. Atelectasis and ventilatory blood flow ratio mismatch due to isolated lung ventilation were avoided, and the improved respiratory mechanics of the prone position did not cause severe hypoxemia.

In addition to the fact that the alveoli were directly exposed to CO_2_ due to the effect of artificial pneumothorax with CO_2_, EtCO_2_ was significantly higher. Complications associated with hypercarboxemia did not occur, but cases with PaCO_2_ ≥ 80–100 mmHg^32^ have been reported, and the safe tolerance range is yet to be determined^33^. Regarding the respiratory setting in this study, we found a tendency to respond to elevated EtCO_2_ by increasing respiratory frequency and decreasing PEEP, suggesting that further increases in airway resistance and shortening of expiratory time may contribute to the high CO_2_ levels.

There are some limitations associated with this study. First, it was a single-center, retrospective study and was not randomized to one-lung ventilation. Some of the ventilator settings were performed at the discretion of the attending anesthesiologist, and there was no strict unification of conditions. With regard to measurements, other respiratory parameters such as Cst, pleural, and transpulmonary pressures were not measured. In addition, the clinical utility and relevance of two-lung ventilation in the prone position compared with those of conventional one-lung ventilation in terms of surgical parameters such as operation time, blood loss, number of mediastinal lymph nodes removed, and long-term postoperative morbidity were not evaluated^34^.

PP-VATS-E in which artificial pneumothorax two-lung ventilation was performed was predicted to be difficult to manage due to worsening of the ventilatory blood flow ratio and alveolar hypoventilation. However, the results of this study showed that the improvement in respiratory mechanics in both the prone position and artificial pneumothorax two-lung ventilation technique did not reduce the oxygenation capacity requiring treatment. This study suggests that in PP-VATs-E, artificial pneumothorax two-lung ventilation is a safe way to manage anesthesia with little negative impact on respiratory mechanics. However, further randomized controlled trials are required to compare pneumothorax two-lung ventilation with the conventional one-lung ventilation, and to assess long-term results.

## Data Availability

The datasets generated during and/or analyzed during the current study are available from the corresponding author on reasonable request.
